# Genome Sequences of three CT cluster Bacteriophages isolated in Durham, North Carolina on *Gordonia rubripertincta*

**DOI:** 10.17912/micropub.biology.001487

**Published:** 2025-02-15

**Authors:** Kalli J. Simmons, Amandine R. Lambert, Marius W. Loekken, Lorelei G. Schreiner, Corey R. Copley, Torrent H. Murthy, Amani S. Nelson, David B. Rosenberg, Adrian L. Vanderputten, Alex Broussard, Kathryn Shriver, Marie P. Fogarty

**Affiliations:** 1 Science and Math, Durham Technical Community College, Durham, North Carolina, USA

## Abstract

PotPie, HippoPololi and BillDoor are bacteriophages with siphoviral morphologies that were isolated from soil in North Carolina using *Gordonia rubripertincta* . The three phages are all grouped in the CT cluster, with genomes of 48182 bp, 45423 bp, and 44875 bp, respectively, and are predicted to be lytic.

**Figure 1. Transmission electron microscopy and plaque morphology of three Cluster CT phages:**
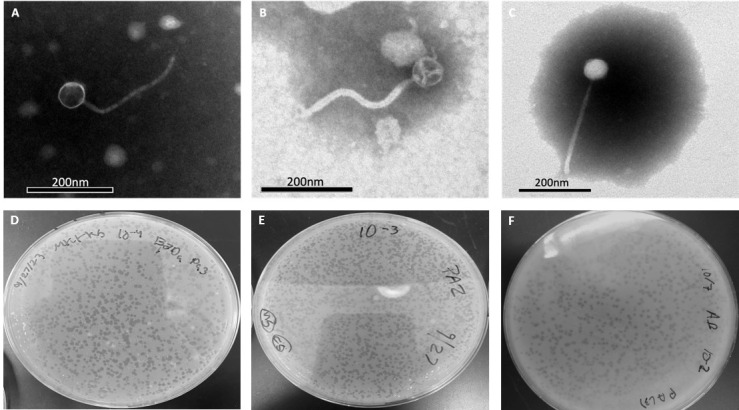
PotPie(A), HippoPololi(B) and BillDoor(C) have siphoviral morphologies, with long and flexible tails. Lysates were negatively stained using 2 % phosphotungstic acid. PotPie(D), HippoPololi(E) and BillDoor(F) form clear plaques.

## Description

Bacteriophages are the most common biological entities on the planet. Coupled with their genetic diversity, they represent an attractive alternative therapeutic against the increased occurrences of antibiotic-resistant bacterial infections (Strathdee et al., 2023) . Here, we report the isolation and genome sequences of three bacteriophages predicted to be lytic.

PotPie, HippoPololi and BillDoor were isolated from soil samples collected in Durham, NC (Table 1). Isolations were performed by shaking soil samples with peptone-yeast calcium (PYCa) liquid medium, filtering through 0.22 µm filters, inoculating the filtrate with *Gordonia rubripertincta* NRRL B-16540 and incubating with shaking at 30˚C for two-five days. An aliquot of the culture was filtered, spotted on PYCa top agar containing *Gordonia rubripertincta* and incubated at 30 ℃ for 3 – 5 days. Bacteriophages were purified through three rounds of plating for plaques before lysates were prepared (Zorawik et al., 2024) . Lysates were negatively stained and imaged by transmission electron microscopy (TEM) to reveal siphovirus morphologies (Table 1, Figure 1 ).

Genomic DNA was isolated from lysates using phenol-chloroform-isoamyl alcohol extraction (Sigma-Aldrich, P2069). Genome sequencing was performed by the Pittsburgh Bacteriophage Institute, using the NEBNext Ultra II FS kit for library preparation and an Illumina MiSeq (v3 reagents) for sequencing, to generate 150 base single-end reads. Sequence reads were assembled using Newbler v2.9 and genomes were checked for accuracy and completion using Consed v.29 (Genivaldo et al., 2013, Gordon and Green, 2013, Russell, 2018). All three phage genomes have 3′ single-stranded overhang ends. The overhang sequences are reported in Table 1, along with number of reads, genome coverage and length, and GC content for each phage.

The genomes were auto-annotated using DNA Master v5.23.6 (Pope and Jacobs-Sera, 2018) . Glimmer v3.02b (Delcher et al., 2007) , GeneMark v2.5p (Besemer & Borodovsky, 2005) and Starterator v1.2 (http://phages.wustl.edu/starterator/) were used to assess start sites and coding potential. Manual inspection and annotation refinement was carried out using PECAAN (discover.kbrinsgd.org), Phamerator using Actino_draft database v578 (Cresawn et al., 2011) , NCBI BLAST searches against the NCBI non-redundant and Actinobacteriophage databases (Altschul et al., 1990) , and HHpred searches against the PDB_mmCIF70, Pfam-A_v36, UniProt-SwissProt-viral70_3, and NCBI_Conserved_Domains(CD)_v3.19 databases (Zimmermann et al., 2018) . Deep TMHMM v1.0.24 was used to detect putative transmembrane domains (Hallgren et al., 2022) , and Aragorn v1.2.41 (Laslett and Canback, 2004) and tRNAscan-SE v2.0 (Lowe and Chan, 2016) were used for tRNA prediction. Default settings were used for all software.

Based on gene content similarity (GCS) of at least 35% to phages in the Actinobacteriophages database, phagesDB (Russell and Hatfull, 2017) PotPie, HippoPololi, and BillDoor are assigned to phage cluster CT. As with other cluster CT phages, genes involved in structure and assembly are located within the first third of the genome and genes involved in lysis, DNA metabolism, and replication are throughout the remaining two thirds of the genome (Pope et al., 2017) . Three transmembrane proteins are encoded directly downstream of the two adjacent lysin A genes in the lysis cassette and may play a role in host lysis (Pollenz et al., 2022) . As observed in other CT cluster phages (McGarrah et al., 2023) , four genes predicted to encode enzymes involved in thymine synthesis were identified. These genes are encoded in an operon-like organization, suggesting that they may work together to support DNA metabolism. As with previously characterized cluster CT phages, no integrase or immunity repressor functions could be identified, suggesting they are unlikely to establish lysogeny.

Data availability

PotPie is available at GenBank with Accession No. PP978854 and Sequence Read Archive No. SRX24892103 . HippoPololi is available at GenBank with Accession No. PP978879 and Sequence Read Archive No. SRX24892096 . BillDoor is available at GenBank with Accession No. PP208920 and Sequence Read Archive No. SRX24892110 .

Table 1: Isolation and sequencing parameters, and phage characteristics

**Table:** 

Parameter	PotPie	HippoPololi	BillDoor
GPS co-ordinates	36.1214 N, 78.72028 W	35.99537 N, 78.88285 W	36.074332 N, 78.917658 W
Plaque size (mm) and Morphology	0.75 - 1 clear	0.8 -1 clear	0.5 – 0.75 clear
Capsid size (nm)	52.2 – 56.7 (n = 4)	58.1-61.5 (n = 4)	61.5 - 63.8 (n = 4)
Tail length (nm)	200 – 218 (n= 4)	266 – 286 (n = 4)	255-276.9 (n=4)
Number of Reads	241648	67479	110912
Average Fold Coverage	709	151	261
Genome Length (bp)	48182	45423	44875
Genome End 3′ single-stranded overhang	5'-CGGCGGTAGGCTT	5'-CGGTAGGCAT	5'-CGGTAGGCTT
GC content %	60.7	61.2	59.7
Gene Content Similarity (GCS)	57.9 % with HippoPololi61.4 % with BillDoor	57.9 % with PotPie67.2% with BillDoor	61.4% with PotPie67.2 with HippoPololi
Number of genes with predicted function / Total predicted genes	38/71	38/68	35 / 69

## References

[R1] Altschul SF, Gish W, Miller W, Myers EW, Lipman DJ (1990). Basic local alignment search tool.. J Mol Biol.

[R2] Besemer J, Borodovsky M (2005). GeneMark: web software for gene finding in prokaryotes, eukaryotes and viruses.. Nucleic Acids Res.

[R3] Cresawn SG, Bogel M, Day N, Jacobs-Sera D, Hendrix RW, Hatfull GF (2011). Phamerator: a bioinformatic tool for comparative bacteriophage genomics.. BMC Bioinformatics.

[R4] Delcher AL, Bratke KA, Powers EC, Salzberg SL (2007). Identifying bacterial genes and endosymbiont DNA with Glimmer.. Bioinformatics.

[R5] Silva GG, Dutilh BE, Matthews TD, Elkins K, Schmieder R, Dinsdale EA, Edwards RA (2013). Combining de novo and reference-guided assembly with scaffold_builder.. Source Code Biol Med.

[R6] Gordon D, Green P (2013). Consed: a graphical editor for next-generation sequencing.. Bioinformatics.

[R7] Hallgren Jeppe, Tsirigos Konstantinos D., Pedersen Mads Damgaard, Almagro Armenteros José Juan, Marcatili Paolo, Nielsen Henrik, Krogh Anders, Winther Ole (2022). DeepTMHMM predicts alpha and beta transmembrane proteins using deep neural networks.

[R8] Laslett D, Canback B (2004). ARAGORN, a program to detect tRNA genes and tmRNA genes in nucleotide sequences.. Nucleic Acids Res.

[R9] Lowe TM, Chan PP (2016). tRNAscan-SE On-line: integrating search and context for analysis of transfer RNA genes.. Nucleic Acids Res.

[R10] McGarrah CEE, Algarin-Martinez ED, Cavasini MED, Correa V, Danielson DF, Dean WR, French JL, Gaskin NN, Jain U, Janvier J, Macumber BM, Martini FK, Mazzei SG, Mujica JM, Odegaard O, Quaterman C, Rand TM, Seidensticker NS, Serrano T, Soltys A, Ungrey MD, Pollenz RS (2023). Isolation and Annotation of Azira, a CT Cluster Phage That Infects Gordonia rubripertincta.. Microbiol Resour Announc.

[R11] Pollenz RS, Bland J, Pope WH (2022). Bioinformatic characterization of endolysins and holin-like membrane proteins in the lysis cassette of phages that infect Gordonia rubripertincta.. PLoS One.

[R12] Pope WH, Jacobs-Sera D (2018). Annotation of Bacteriophage Genome Sequences Using DNA Master: An Overview.. Methods Mol Biol.

[R13] Pope WH, Mavrich TN, Garlena RA, Guerrero-Bustamante CA, Jacobs-Sera D, Montgomery MT, Russell DA, Warner MH, Hatfull GF, Science Education Alliance-Phage Hunters Advancing Genomics and Evolutionary Science (SEA-PHAGES) (2017). Bacteriophages of Gordonia spp. Display a Spectrum of Diversity and Genetic Relationships.. mBio.

[R14] Russell DA (2018). Sequencing, Assembling, and Finishing Complete Bacteriophage Genomes.. Methods Mol Biol.

[R15] Russell DA, Hatfull GF (2017). PhagesDB: the actinobacteriophage database.. Bioinformatics.

[R16] Strathdee SA, Hatfull GF, Mutalik VK, Schooley RT (2023). Phage therapy: From biological mechanisms to future directions.. Cell.

[R17] Zimmermann L, Stephens A, Nam SZ, Rau D, Kübler J, Lozajic M, Gabler F, Söding J, Lupas AN, Alva V (2017). A Completely Reimplemented MPI Bioinformatics Toolkit with a New HHpred Server at its Core.. J Mol Biol.

[R18] Zorawik M, Jacobs-Sera D, Freise AC, Reddi K, SEA-PHAGES (2024). Isolation of Bacteriophages on Actinobacteria Hosts.. Methods Mol Biol.

